# Measurements on the external load acting on aquatic resistance fins during flexion/extension movements using a robotic joint

**DOI:** 10.3389/fphys.2022.1046502

**Published:** 2022-12-02

**Authors:** M. K. Gislason, I. T. Einarsson, S. S. Ingvason, J. M Saavedra, B. Waller

**Affiliations:** ^1^ School of Engineering, Department of Biomedical Engineering, Reykjavik University, Reykjavik, Iceland; ^2^ Physical Activity, Physical Education, Sport and Health Research Centre (PAPESH), Sports Science Department, School of Social Sciences, Reykjavik University, Reykjavik, Iceland

**Keywords:** aquatic resistance training, load measurements, biomechanics, robotic arm, instrumentation, aquatic rehabilitation

## Abstract

Aquatic resistance training has been proven to be beneficial to many people, in particular those struggling with degenerative joint diseases or recovering from other musculoskeletal issues as the reaction forces acting on the joints become lower, but without compromising the cardiovascular and neuromuscular benefit of the movement. Little has been written on the load produced by or measurements of the devices used in aquatic resistance training. Therefore, uncertainties exist regarding details of how much load can be applied onto the foot when performing the movements and how to quantify progression. In this study, an instrumented robotic arm was designed, built, and used to measure the load acting on the three different types of fins during a simulated flexion/extension movement of a knee. The angular velocities of the knee ranged from 25°/s to 150°/s, which represent the physiological range of *in vivo* movements. The results demonstrated that the load followed a second-order polynomial with the angular velocities. The load is therefore a function of the angular velocity, the surface area of the fins, and the location of the fins away from the joint center rotation. We modeled the progression of speeds at maximal voluntary movements based on previous studies. The maximum loads measured between 11 kg and 13 kg in extension and 6 kg and 9 kg in flexion at 150°/s rotational velocity.

## Introduction

Aquatic exercise is effective in improving function and decreasing pain in people with musculoskeletal conditions, such as osteoarthritis, and after joint replacement surgery (Valtonen et al., 2010; Bartels et al., 2016) or as a fall prevention intervention in elderly people (Ferreira et al., 2020). While aquatic exercise is a popular training method, there are limitations in the tools available to qualify the work done by the person, especially in terms of the work performed by the musculoskeletal system. Currently, there are no known methods to directly quantify the external resistance imparted on the moving limb and thus work for the muscles e.g., the work done by the quadriceps during knee extension, when compared, for example, to easily measurable and adjustable external load in a weights machine in the gym. This makes the prescription of progressive resistance training exercises difficult. This may explain the results from a recent systematic review indicating that aquatic exercise does not improve muscular strength in people with musculoskeletal (MSK) conditions (Waller et al., 2013; Heywood et al., 2017). There is no evidence that immersion inhibits force production during voluntary muscle activation (Pöyhönen et al., 2001a), which does not explain the lack of improvements in muscle strength seen in the aquatic environment alone.

However, the studies included in the previously mentioned reviews only included aquatic exercises conducted without additional external resistance devices. Barefoot training in water, at maximum voluntary velocity, has been estimated to produce only 33% of knee extension power, which may not be sufficient to stimulate strength improvements (Pöyhönen et al., 2001b). However, maximum velocity was not part of the exercise prescription in most of the included studies looking at the effect of aquatic exercises on muscle strength in people with MSK conditions (Waller et al., 2014; Heywood et al., 2017). Further, it was shown that up to 85% maximum voluntary contraction (MVC) of the muscle was achieved in male and female subjects using external resistance devices (Pöyhönen et al., 2001a). This could be due to the physical properties of water. When an object is moving through the water it is exposed to “drag forces” that impart an external force (F) on the object.

$$F=\frac{1}{2}C_{d}\rho Av^{2}$$

In the above equation, ρ is the fluid density, v is speed through the fluid, A is the object’s surface area, and $C_{d}$ is the relevant drag coefficient, which depends on the shape of the object relative to the direction of flow. High-quality randomized controlled studies have shown that aquatic resistance training (ART) improves muscle power and muscle mass in healthy older adults (Pöyhönen et al., 2002) and in patients after total knee replacement surgery (Valtonen et al., 2010). This indicates that the addition of devices to increase surface area and the relative drag coefficient are essential aspects of optimizing external load in aquatic exercise.

Velocity and the object’s surface area are two variables that can be controlled during ART with the addition of external resistance devices. However, there is confusion over whether an individual variable or a combination of variables is the most important to manipulate in order to prescribe progressive aquatic strength training programs. Further, to date, no study has described the additional load an aquatic resistance device imparts around the moving joint. Therefore, the aim of this study was to objectively and directly measure the additional external load produced by different aquatic resistance devices. Additionally, this study investigated the direct effect that changing velocity and surface area had on the external load.

## Methods

The experimental design was based on creating a measurement system capable of capturing the load acting on resistance fins and to be able to export the data. An instrumented robotic arm was built at Reykjavik University to measure the additional load created by different aquatic resistance devices. First, an arm was constructed which consisted of 2 bars connected *via* a hinge joint powered by a three phase motor (Bauser DGK 6550). The velocity of the motor was controlled using an Altivar 18 speed drive controller for motors. Finally, a structural frame of steel was welded onto a water tank (2.0 × 2.0 × 1.5 m) to secure the arm during movement. A counterweight was placed on the frame in order to maintain the stability of the motor.

**FIGURE 1:**
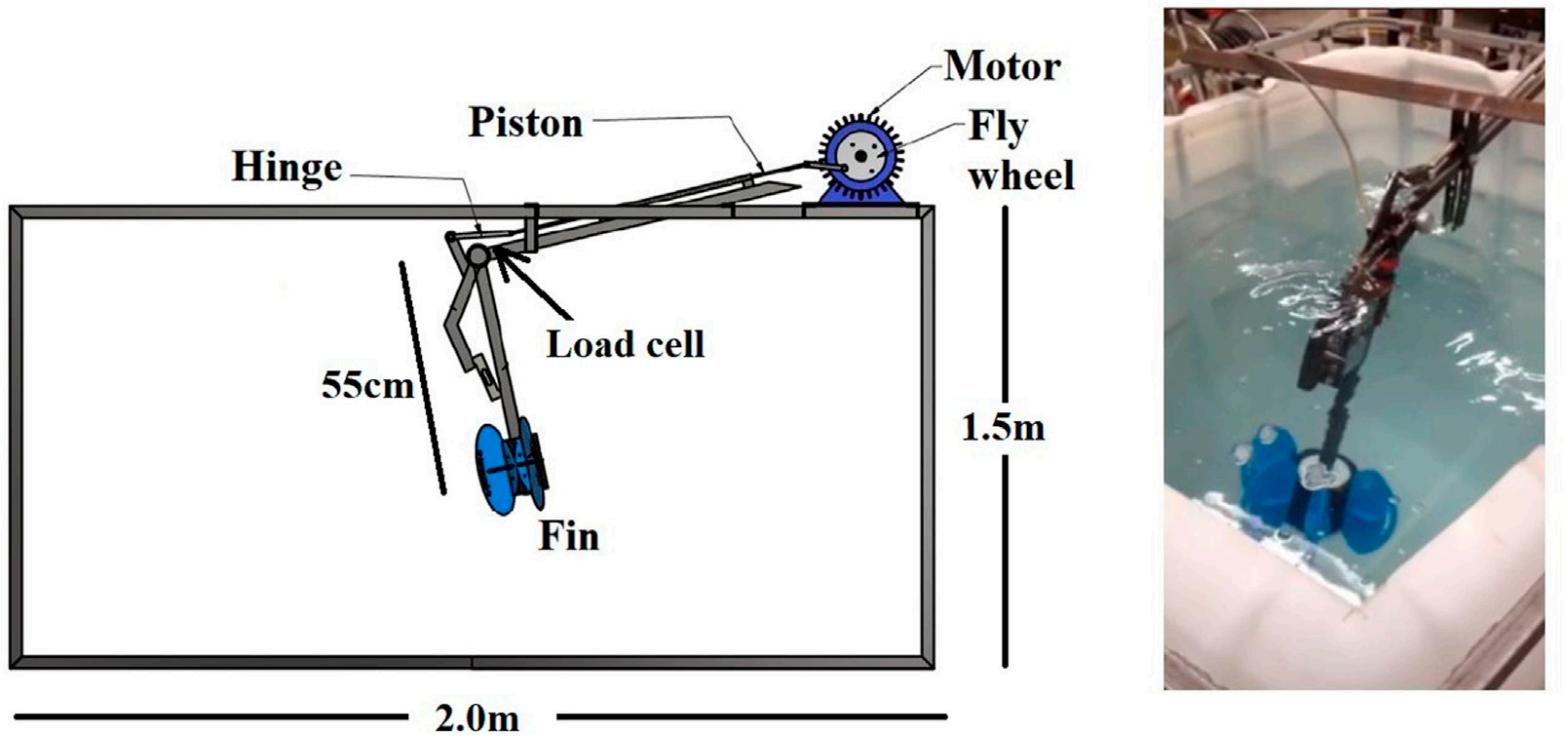
Setup of the measurement system.

An image of the setup can be seen in Figure 1.

At the distal end of the arm, a plastic cylinder was placed on which the aquatic fins were positioned. The bottom of the fin was placed in line with the bottom of the arm, to reproduce as accurately as possible the position of the talar joint during normal use. The distance from the hinge joint to the distal end of the arm was 55 cm.

A load cell (Vishay 1510) was placed at the hinge joint to measure the external load it was subjected to during movement and was synchronized with an electric goniometer that read the flexion/extension angle of the robotic arm. The synchronization was carried out using an Arduino microcontroller. The load measurements were read into a computer using a bridge input module from National Instruments (NI-9237). As the load was measured at the hinge joint, the extension loads were positive and flexion loads were negative. Loads were scaled according to the distance from the hinge joint to the center of pressure of each fin.

**FIGURE 2:**
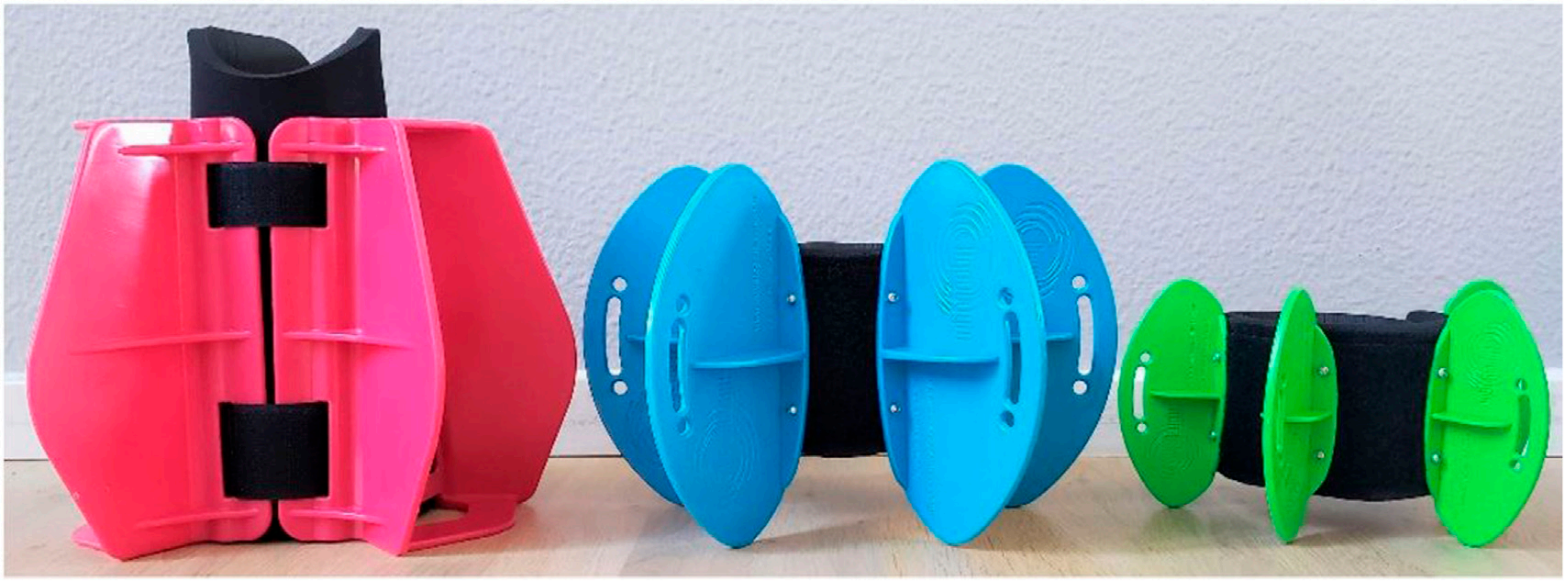
Shows the types of leg resistance fins tested.

Three types of leg fins were tested and can be seen in Figure 2.

The fins and their cross sectional area tested were (see Figure 2):

• Pink fin: Lower limb fins from Aquastrength/Aqualogix (400 cm^2^)
• Blue fin: Max resistance fins from Aqualogix (350 cm^2^)
• Green fin: High speed hybrid fins from Aqualogix (140 cm^2^)

Each fin was tested in the water over a range of velocities that were controlled manually using the motor controller. The angle and load as a function of time were logged at 10 Hz.

## Results

**FIGURE 3:**
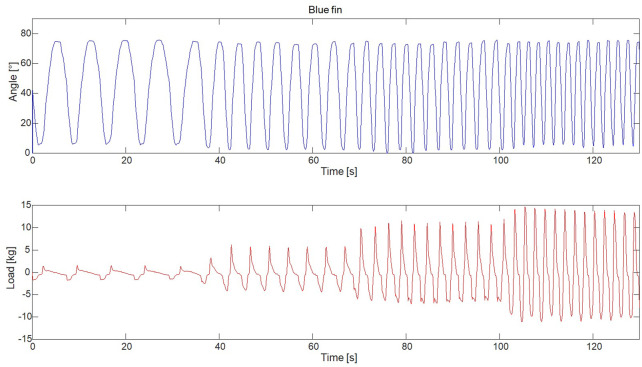
Shows the raw measured data of the joint angles as a function of time.

An example of the measurement output is seen in Figure 3, demonstrating the measured joint angles and load as a function of time. Figure 3 indicates how the velocity of the flexion/extension movement of the robotic arm was continuously increased, ranging from 20°/s to 150 °/s. Since the velocity was changed on the motor itself, which had a fixed torque at each rotational velocity, no direct control was imparted on the joint rotation angular velocity on the arm itself. The upper limit of the velocity of the robotic arm was at 150°/s as the structure started to shake and became unstable

**FIGURE 4:**
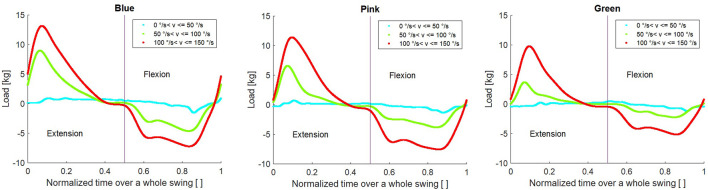
Shows the load cycle over a normalized swing for each of the fins for different set of velocities.

Each cycle, which constituted of going to full extension and back again, was identified and isolated. The load was then plotted as a function of the normalized time. The results can be seen in Figure 4.

From the figure, it can be seen how the maximum load comes relatively early in the swing phase, going from 90° flexion (at t = 0) to almost full extension (at t = 0.5) and back to 90° flexion (at t = 1) to start the next swing phase.

**FIGURE 5:**
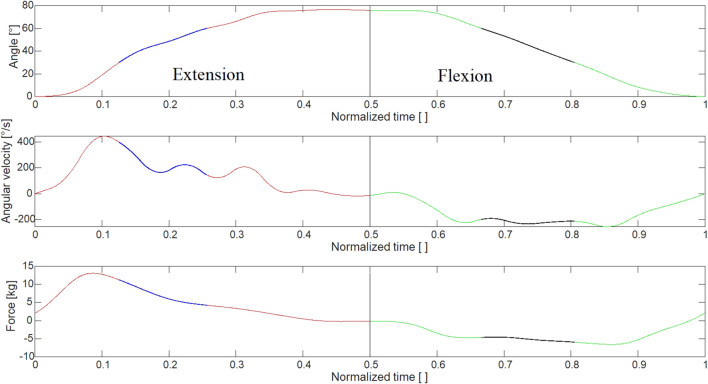
Single cycle showing the region where the average load is acting within the swing during extension of the knee (blue) and flexion (black).

Finally, each swing phase was analyzed, as can be seen in Figure 5. The average load acting during the extension and flexion phase was identified by looking at the loads during mid-swing ranging from 30° to 60°. The regions defined can be seen in Figure 4. The average loads were calculated for this region. This region was selected as the impact loads from the direction changes had subsided and the load curve became more uniform within this region.

For each swing phase, the average and the maximum loads were collected for both extension and flexion. They were then plotted as a function of the calculated angular velocity within the region, obtained by differentiating the time-angle curve. As can be seen from the figure, the angular velocity was not a constant during the swing.

Each curve was fitted with a second order polynomial, using the following model:

$$F=\frac{A*v^{2}+B*v+C}{1000}$$

As we assume that no force is applied when there is no movement, the polynomials are constrained so that they must go through (0,0) and therefore C = 0.

The coefficients of each polynomial calculating the maximum force during theload cycle can be seen in Table 1.

Statistical analysis was carried out for the load acting on the three different fins at rotational velocities between 100°/s and 150°/s for both flexion and extension. A *t*-test was used to compare the maximum load values at the velocity interval between the three different types of fins. The results can be seen in Table 2.

**TABLE 1:** Coefficients of the fitted polynomials.

	Extension		Flexion	
	A $\left[\frac{kgs^{2}}{m^{2}}\right]$	B $\left[\frac{kg\;s}{m}\right]$	A $\left[\frac{kgs^{2}}{m^{2}}\right]$	B $\left[\frac{kg\;s}{m}\right]$
Blue fin	0.798	54.131	0.383	24.473
Green fin	0.698	-2.554	0.192	7.842
Pink fin	0.697	25.423	0.276	26.564

**TABLE 2:** Average maximum load at high velocities for both flexion and extension.

	Extension		Flexion	
	Maximum load [kg]		Maximum load [kg]	
	**Average**	**Std**	**Average**	**Std**
Blue fin	13.3	0.3	7.7	1.0
Green fin	10.0	1.3	4.2	1.0
Pink fin	11.8	0.5	7.8	0.8

There was a strong statistically significant difference between the maximum load between all fins for extension (*p*-values of the order of magnitude $10^{-7}$ ) as well as in flexion, apart from the blue fin vs. the pink fin (*p*-value 0.78).

## Discussion

Our unique method of measuring load created during aquatic exercise was the first to directly objectively measure the torque created around a joint and convert it into load acting on the resistance fin. This method showed the expected results of a larger surface area and higher angular velocity that increase the forces in a polynomial progression. This is the first study, to our knowledge, to directly measure the forces created by aquatic resistance devices using a velocity-controlled motor to drive an instrumented robotic arm. The system is able to recreate a movement that is comparable to that of a human one at physiologically relevant rotational velocities.

Our results indicate that at low speeds $\left(<100\;\deg/s\right)$ , there is very small load applied to the fins; therefore to generate loads that are equivalent to those generated on land and to create a training effect, it is essential to rotate the knee at a higher rate.

The results indicate how the load is not uniform during either flexion or extension. For the extension, a peak force can be seen between 5 and 10% of the whole cycle depending on the load rate. This peak is generated from the impulse due to the change in movement and can also be seen when the movement transitions from extension to flexion. This is thought to be an effect of moving an object in the opposite direction of the moving water, caused by the reciprocal movement. However, the force values are somewhat lower in flexion and force plateaus between 60 and 90% of the movement. The peak forces are also in line with the work from Kutzner et al. (2017) who showed that peak compressive forces, as measured with instrumented joint implants, occurred at the point of changing direction in aquatic exercises. Additionally, this peak force is seen at the end of the acceleration phase from stationary, and it has been suggested that acceleration and not just constant velocity is also important for maintaining and increasing drag resistance. This indicates that quick and small repetitive movements with attempts to maintain acceleration i.e., trying to go faster, would be favored to optimize the tension and thus load on the muscle. This contrasts with the slow rhythmical movements that focus on achieving a full range of motion as discussed in the majority of published studies looking into the effects of aquatic exercise on muscle strength.

The angular velocities reached during this experiment were up to three times slower than those measured during barefoot knee extension by (Pöyhönen et al., 2001a; Pöyhönen et al., 2001b). Study indicated that healthy adults could produce maximal angular velocities of values between 364°/s and 473°/s. In line with this data, the peak velocities seen in our study in the robotic arm were in the region of 400°/s, however these were just instantaneous velocities achieved without external resistance and not representative of physiological average rotation velocity values. Based on the extrapolation, load at velocities in the region of 400°/s would result in load values ranging from 70 to 95 kg; however, with additional fins, the maximum angular velocity achieved by healthy adults was approximately 35% slower. As the interpolation is based on the average velocity over a subregion of the flexion and extension movement, looking at other regions of the flexion and extension phase would change the coefficients of the polynomial and therefore the load predictions. Although the speed of the angular velocity was at levels lower than can be achieved at maximal effort during aquatic exercise, our results showed a robust second-order polynomial form which can be used to estimate forces during aquatic exercise by inserting the target angular velocity in [°/s ].

**FIGURE 6:**
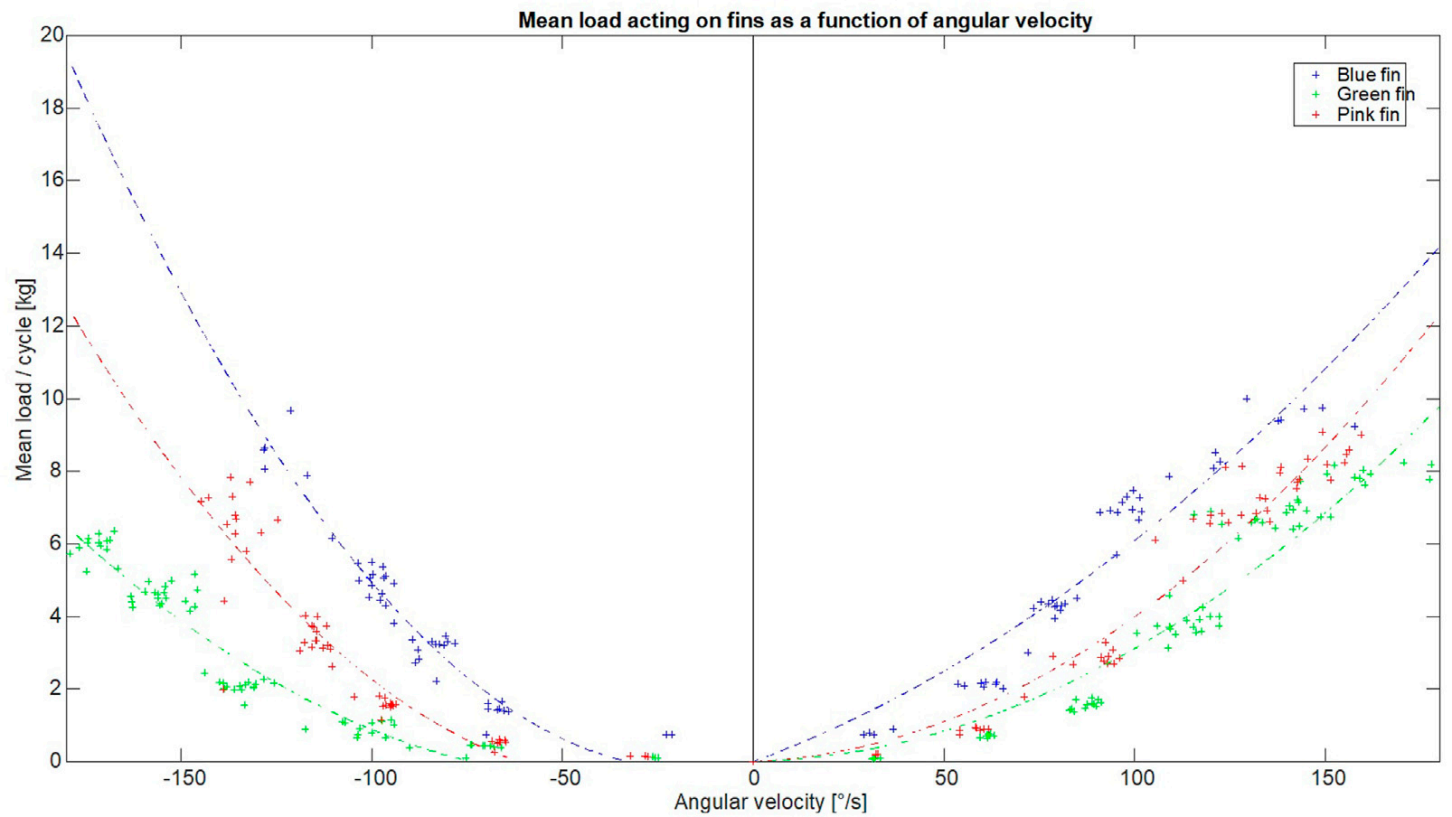
Shows the average load from each swing as a function of calculated angular velocity.

It has been suggested that, in addition to higher angular velocities, increasing surface area would increase the load experienced around the moving joint and could be used to progress exercise prescription of aquatic resistance training, as demonstrated in other studies (Pöyhönen et al., 2001a; Pöyhönen et al., 2001b). Our results initially support this, with the green fin demonstrating statistically lower load values than were found from the blue and pink fins (Figure 6). This can be attributed to the relatively small surface area compared to the other two fins and supports the suggestion that there is an effect in increasing surface area. However, our results also partly contradict this, with the blue fin creating higher additional loads than the pink fin, even though the cross-sectional area is 12.5% smaller. In addition to the surface area, there are several aspects regarding fin design that could explain this difference. Firstly, the center of pressure in the pink fin is higher in design than the blue fin, with the blue fin’s center of pressure extending lower, theoretically past the talar joint. The lever arm was estimated to be 5 cm longer for the blue fins, potentially explaining some of the differences. Secondly, the design of the blue and pink fins are different. Each fin consists of four blades to allow multiplane movements, however the blades in the blue fin are more mobile, while the pink are rigidly fixed together. The possibility should not be excluded that this may result in a different attack angle during movement, changing the amount of turbulent flow around the fin and resulting in changes of drag resistance, thus affecting the loading.

The duration for which a muscle is under tension during a contraction as well as an external load is important for optimal adaption to training (Burd et al., 2012). Therefore, maximizing the duration and force produced in this phase during a training set could be essential for optimal physiological response. Our results demonstrate that the sustained force produced by the fins is generated during the mid-portion (3/5th) of the flexion and extension movement for all fins and velocities over 100°/s, as shown in Figure 4 and Figure 5. It could be hypothesized that while the smaller fin creates less force, it can be moved at a higher frequency and could possibly create a similar overall loading in the muscle when compared to larger fins. For example, the angular velocity needed to reach 25 kg of force would need to be 191°/s and 239°/s for blue and green fins respectively. Using repetition data from a previous study (Waller et al., 2017) which recruited women with mild knee osteoarthritis, the maximum range of average angular velocity for knee extension/flexion in sitting was calculated as 213°/s using large resistance devices and 240°/s using small resistance devices. Therefore, the required velocities are within feasible physiological movement range in a population who is often recommended aquatic exercise. Further, when looking at overall load and applying our results to a typical training set of 45 s for aquatic resistance training (Pöyhönen et al., 2002; Valtonen et al., 2010; Waller et al., 2017), the difference in overall load between fins is 3% (estimating repetition completed in 45 s is 38 with blue fins and 46 with green). Therefore, this supports the idea that sustained velocity during training may be more important than increasing surface area, which is in agreement with the fact that the velocity is squared in the equation for calculating drag forces. However, the ability of a muscle to recruit muscle units quickly enough may be a barrier to generating sufficient angular velocity to achieve this calculated training effect in many deconditioned populations. This hypothesis requires testing in future research, which should investigate if baseline muscle strength and power predict which type of aquatic resistance training parameters create an optimal training stimulus *in vivo*.

## Limitations

The velocity of the robotic arm was controlled *via* the motor, whose torque output was a constant; therefore the angular velocity of the simulated knee was not a constant throughout the movement, resulting in possible jerks that could influence the load readings. Additionally, the maximum velocity obtained with the arm was somewhat lower than the ones that people could reach in the pool. This was due to the instability of the test rig and the motor when reaching those velocities. We only measured the resistance of the device alone and measured the external forces, and so the behavior of the fin could be different once attached above the ankle.

### Practical applications

Using a platform like the one demonstrated in this study could provide an objective standardized assessment of the overall load that is acting on the fin during rotational movements of the leg. Such a model can then be used as a guideline regarding the speed of the movement carried out by the patient in the pool, which could be controlled using a metronome or similar device, and will provide the clinician with an estimate of the external load acting on the leg during the movement.

## Conclusion

The methodology showed the method of measuring torque created during aquatic exercise. The results were as expected and indicate the potential of developing further modelling of forces created during aquatic exercise. Our results indicated that progression of additional external forces can be estimated using surface area and/or angular velocity. Future studies should focus on investigating how different devices interact with voluntary muscle activation *in vivo* as well as how lower limb size and size of the fin affect the training parameters.

## Data Availability

The raw data supporting the conclusions of this article will be made available by the authors, without undue reservation.
