# Supplementary material for: Measurements on the external load acting on aquatic resistance fins during flexion/extension movements using a robotic joint
Source: Front Physiol. 2022 Dec 2;13:1046502. doi: 10.3389/fphys.2022.1046502 (PMC9755156; doi:10.3389/fphys.2022.1046502)
Supplement: Supplementary file 2 [file Table1.DOCX]

## Supplementary material 1: Estimated forces at training speeds

Using repetition data from our previous study we can estimate the progressive external force created by the different devices at different speeds for during seated knee flexion and extension (Waller et al., 2017). In our study, women with mild knee osteoarthritis aged 60-68 years old, we record the number of full repetitions completed during three sets of 45 seconds of knee flexion and flexion. This was also completed in three different conditions i.e., barefooted, with small resistance devices (surface area 181cm^2^) and large resistance devices (750cm^2^). Average repetitions for 45 seconds were 40, 32 and 28 for barefoot, small and large devices respectively. We estimated the equivalent velocity as that performed by the robotic arm as 213, 170 and 149 deg/s for barefoot, small and large devices respectively. Maximum repetitions and speed where 50 (267 deg/s), 45 (240deg/s) and 40 (213 deg/s) for barefoot, small and large respectively.

Figures 1 and 2 below show the range of possible repetition rate where maximum repetition speed would be approximate 1.1 repetitions of knee flexion and flexion per second (270deg/s). Interestingly, when comparing the green fins at 240 deg/s the forces created are like those created by the blue fin at 190 deg/s. Force calculator can be found from supplementary material 2. These figures are assuming the distance to center of pressure is the same, however, the different between center of pressure for the blue and pink devices is approximately 5 cm lower than the pink.

Figure 1 Forces created by the different resistance devices at different angular speeds during extension

Figure 2 Forces created by the different resistance devices at different angular speeds during flexion

**Reference**

Waller, B., Munukka, M., Multanen, J., Rantalainen, T., Pöyhönen, T., Nieminen, M. T., ... & Heinonen, A. (2013). Effects of a progressive aquatic resistance exercise program on the biochemical composition and morphology of cartilage in women with mild knee osteoarthritis: protocol for a randomised controlled trial. *BMC musculoskeletal disorders*, *14*(1), 1-14.
